# An Outbreak of Severe Infections with Community-Acquired MRSA Carrying the Panton-Valentine Leukocidin Following Vaccination

**DOI:** 10.1371/journal.pone.0000822

**Published:** 2007-09-05

**Authors:** Tang Chi Thuong, Nguyen Dac Tho, Ngo Thi Hoa, Nguyen Thi Minh Phuong, Le Van Tuan, To Song Diep, Jodi Lindsay, Nguyen The Dung, Bach Van Cam, Le Quoc Thinh, Le Thanh Hai, Le Dieu Linh, James Campbell, Nguyen Thi Kim Tien, Nguyen Van Vinh Chau, Joshua Cockfield, Le Truong Giang, Phan Van Nghiem, Le Hoang Son, Huynh Tan Son, Le Van Phung, Megan Counahan, Adwoa Bentsi-Enchill, Richard Brown, James Simmerman, Nguyen Tran Chinh, Tran Tinh Hien, Jeremy Farrar, Constance Schultsz

**Affiliations:** 1 Pediatric Hospital No. 1, Ho Chi Minh City, Viet Nam; 2 Preventive Medicine Center, Ho Chi Minh City, Viet Nam; 3 Oxford University Clinical Research Unit, Hospital for Tropical Diseases, Ho Chi Minh City, Viet Nam; 4 Pasteur Institute, Ho Chi Minh City, Viet Nam; 5 World Health Organization, Ho Chi Minh City, Viet Nam; 6 Hospital for Tropical Diseases, Ho Chi Minh City, Viet Nam; 7 St. George's Hospital, London, United Kingdom; 8 Health Services of Ho Chi Minh City, Ho Chi Minh City, Viet Nam; 9 National Institute for Control of Vaccines and Biologicals, Hanoi, Viet Nam; University of Liverpool, United Kingdom

## Abstract

**Background:**

Infections with community-acquired methicillin-resistant *Staphylococcus aureus* (CA-MRSA) are emerging worldwide. We investigated an outbreak of severe CA-MRSA infections in children following out-patient vaccination.

**Methods and Findings:**

We carried out a field investigation after adverse events following immunization (AEFI) were reported. We reviewed the clinical data from all cases. *S. aureus* recovered from skin infections and from nasal and throat swabs were analyzed by pulse-field gel electrophoresis, multi locus sequence typing, PCR and microarray. In May 2006, nine children presented with AEFI, ranging from fatal toxic shock syndrome, necrotizing soft tissue infection, purulent abscesses, to fever with rash. All had received a vaccination injection in different health centres in one District of Ho Chi Minh City. Eight children had been vaccinated by the same health care worker (HCW). Deficiencies in vaccine quality, storage practices, or preparation and delivery were not found. Infection control practices were insufficient. CA-MRSA was cultured in four children and from nasal and throat swabs from the HCW. Strains from children and HCW were indistinguishable. All carried the Panton-Valentine leukocidine (PVL), the staphylococcal enterotoxin B gene, the gene complex for staphylococcal-cassette-chromosome *mec* type V, and were sequence type 59. Strain HCM3A is epidemiologically unrelated to a strain of ST59 prevalent in the USA, although they belong to the same lineage.

**Conclusions:**

We describe an outbreak of infections with CA-MRSA in children, transmitted by an asymptomatic colonized HCW during immunization injection. Consistent adherence to injection practice guidelines is needed to prevent CA-MRSA transmission in both in- and outpatient settings.

## Introduction

*Staphylococcus aureus* is a commensal of the anterior nares in 25% of the human population, and carriage is a risk factor for infection and transmission of *S. aureus* in hospitals and the community [1], [2]. While methicillin resistant *S. aureus* (MRSA) are widespread in hospitals, community-acquired MRSA (CA-MRSA) is increasingly responsible for severe skin and soft tissue infections, necrotising fasciitis, and a fatal form of necrotising pneumonia, in previously healthy individuals [3]–[5]. Currently, CA-MRSA are responsible for 15–74% of skin and soft-tissue infections among patients presenting to emergency departments in the USA [6].

CA-MRSA are often from unique *S. aureus* lineages, and carry additional or variant virulence and resistance genes on mobile genetic elements (MGE). The toxin Panton-Valentine leukocidin (*PV-luk*) and a small variant of the methicillin-resistance cassette (SCC*mec* types IVa, IVc and V), encoding methicillin-resistance but not multi-drug resistance, are typical. CA-MRSA have evolved independently from MRSA found in hospitals. Analysis of 117 CA-MRSA isolates from three continents suggests the spread of a limited number of clones, which are associated with a particular geographic origin but may have originated from methicillin-sensitive *S. aureus* clones which are prevalent on different continents [7], [8].

Infections with CA-MRSA typically occur in clusters in settings with multiple and prolonged close contacts between individuals, such as families, schools, hospital nurseries and sport teams, or in situations were hygiene is poor, such as in jails and facilities for homeless persons [2], [9]–[13]. Here we report an outbreak of severe infections with CA-MRSA, including one with a fatal outcome, occurring within two weeks among nine children in Ho Chi Minh City (HCMC), Viet Nam in May 2006. The epidemiological and microbiological investigations indicate these infections were due to transmission of CA-MRSA by a single health care worker during routine vaccination practice.

## Methods

### Epidemiological and clinical investigations

A field epidemiological investigation was initiated following reports of an outbreak of serious adverse events following immunization (AEFI), involving six children who were admitted to the Pediatric Hospital Number One between 8 and 10 May 2006. Potential cases of AEFI were searched using the vaccination records of the community health centres. Face-to-face interviews with the parent/guardian of affected children and hospital and community health centre staff were completed. A review of vaccination settings and procedures was carried out through a series of visits to vaccination sites, review of records and interviews with vaccination staff and supervisors. Clinical data were obtained by review of the medical records of all cases. This outbreak investigation was initiated and approved by the Health Service of Ho Chi Minh City. Parents or guardians and health care workers and their family members gave informed verbal consent before entry in the investigation.

### Microbiological investigations

Pus was collected from abscesses by needle puncture or from necrotic material using a sterile swab, and cultured using standard culture methods. Blood cultures were performed on admission before administration of antimicrobial therapy, using the BACTEC 9050 continuous monitoring blood culture instrument (BectonDickinson, NJ, USA). Cerebrospinal fluid cultures were not performed.

Screening for carriage of MRSA was performed on 19 May 2006, after clinical samples became positive with MRSA. Trained laboratory staff collected cotton swab samples (Transwab, MW&E, Corsham, UK) from anterior nares, throat and perineum of two vaccinators (vaccinators X and O), two examining medical officers and the person responsible for vaccine storage, all of whom were directly involved in the handling or administration of vaccines. In addition, the mothers of three children (Child 2, 4 and 9 in Table 1) were screened on the same day. Vaccinator X and all her household members (husband, daughter, son) were screened for MRSA carriage on 23 June 2006. Vaccinator X was screened again on 7 September 2006. Swab samples were inoculated onto sheep blood agar (BA), mannitol salt agar containing oxacillin (2 mg/l) (OMSA) and placed in Mueller Hinton broth, within one hour after collection. Plates were incubated for 24–48 hours at 35°C and the broth was incubated for 24 hours and subcultured onto BA and OMSA.

**Table 1:** Clinical characteristics and laboratory values of children on admission, and outcome

Variable*	Child 1	Child 2	Child 3	Child 4	Child 5	Child 6	Child 7	Child 8	Child 9
Age (months)	5	13	13	17	13	14	5	16	15
Sex	Male	male	male	male	male	female	female	female	Male
Date of vaccination Time (hrs)	28 April 08.00	4 May 08.30	8 May 09.00	8 May 10.30	9 May 08.00	9 May 08.30	9 May 08.30	9 May 09.30	10 May 08.30
Time of onset symptoms† (hrs)	9 May	11.30	12.00	13.30	09.00	11.30	19 May	14.00	10.00
Date of in-patient hospital admission	12 May	9 May	9 May	8 May	10 May	9 May	10 May	10 May	10 May
Length of hospitalization (days)	1+16 (re-admission)	12	63	24	<1	8	12	8	14
Vaccine type‡	HBV	MMR	MMR	MMR	MMR	MMR	HBV	varicella	MMR
Swelling at injection site§	+	+	+	+	+	+	+	−	+
Abscess at injection site	+	+	−	-necrosis	−	−	+	−	−
Skin rash or severe erythema	−	local	generalized	generalized	arm, shoulder, left thorax	−	−	generalized	Local
Vomiting	−	−	−	−	−	+	−	−	−
Diarrhea	−	−	+	−	+	+	−	−	−
Max. temp. (°C)	38.0	39.2	41.0	41.0	41.0	39.0	NA	39.5	38.0
Inotropes∥ (days)	−	−	+(7)	+(4)	+(until death)	−	−	−	−
Respiratory support (days)	−	−	+(5)	+(2)	+(until death)	−	−	−	−
Hemoglobin (g/L)	114	101	122	145	150	124	131	112	120
Leukocyte count (10^9^/L)	35,4	23,8	19	16,9	12	11	9,1	14,1	11,2
Percentage neutrophils	72	51	60	61	72	72	44	50	54
Platelet count (10^9^/L)	779	469	210	206	243	201	701	448	241
ALT level (U/l)	NA	NA	84	279	148	24	NA	NA	19
AST level (U/l)	NA	NA	178	253	165	38	NA	NA	32
CK level (U/l)	NA	NA	2,191	14,540	1,952	NA	NA	NA	NA
Serum creatinine (µmol/L)	NA	35	123	115	80	27	NA	NA	35
Blood culture	_	_	_	_	_	_	_	_	_
Pus culture	MRSA**	MRSA**	NA	MRSA**	NA	NA	MRSA**	NA	NA
Antimicrobial treatment	oxacillin, gentamicin	vancomycin, ciprofloxacin	vancomycin, imipenem, metronidazol	vancomycin imipenem metronidazol	oxacillin, vancomycin, cefotaxim, cipofloxacin	vancomycin, ciprofloxacin	oxacillin	oxacillin, cefotaxim	vancomycin, ciprofloxacin
outcome	recovered	recovered	neurological sequelae	recovered	died	recovered	recovered	recovered	recovered

† Date of vaccination unless otherwise stated.

‡ HBV: Hepatitis B virus. Both received Euvax B™; MMR: Measles, Mumps, Rubella. All received Priorix™; varicella: Varilrix^R^.

§ swelling at injection site beyond the expected after routine vaccination.

∥ Inotropes used were dopamine, dobutamine, noradrenaline.

** MRSA: methicillin-resistant *Staphylococcus aureus*. NA denotes not available, a plus sign positive or present, and a minus sign negative or absent.

* Normal ranges are as follows: hemoglobin concentration, 105–135 g/L; leukocyte count, 6 to 17.5·10^9^/L; platelet count, 150 to 400·10^9^/L; alanine aminotransferase (ALT) level, below 37 U/L; aspartate aminotransferase (AST) level, below 40 U/L; creatine phosphokinase (CK) below 200 U/L; serum creatinine concentration, 18–80 µmol/L;

*S. aureus* was identified using catalase, slide coagulation and DNAse tests. Susceptibilities to eight antimicrobial agents (oxacillin, erythromycin, clindamycin, rifampin, ciprofloxacin, trimethoprim-sulfamethoxazole, gentamicin and vancomycin) were tested by disk diffusion test according to CLSI standards [14]. MIC for oxacillin was determined using E-test (AB-Biodisk, Solna, Sweden) on Mueller Hinton agar without NaCl.

Molecular genotyping was performed by pulse field gel electrophoresis (PFGE) with *Sma*I digestion [15]. Genetic relatedness was assessed using the criteria established by Tenover et al. [16]. Multi locus sequence typing (MLST) was performed as described by Enright et al. [17]. Sequence type (ST) assignment was based on the sequence of the alleles at each locus of seven housekeeping genes included in the MLST scheme, using the MLST database (www.mlst.net). MLST results were analyzed using the program eBURST (http://eburst.mlst.net) on the entire MLST database, to assign the ST to its clonal complex [18]. Characterization of the staphylococcal cassette chromosome *mec* elements (SCC*mec*) was performed by PCR using the primers as described by Chongtrakool [19]. The presence of 22 specific staphylococcal virulence genes was determined by PCR [20]. Amplicons were sequenced to confirm the identity of the PCR products.

A recently developed microarray, containing PCR products representing every predicted open reading frame present in seven whole-genome sequences of *S. aureus* (strains MRSA 252, N315, Mu50, COL, 8325, MW2, MSSA476) was used for further analysis [21]. DNA of strains HCM3A and HT2001-0751 was extracted using a genomic DNA extraction kit (EdgeBioSystems, Gaithersburg, MD, USA), labeled and hybridised to the array, and data were analyzed as described previously [21]. Strain HT2001-0751 (SCC*mec* IV-MRSA-ST59), was provided by Dr. J. Etienne, Lyon, France.

## Results

**Figure 1:**
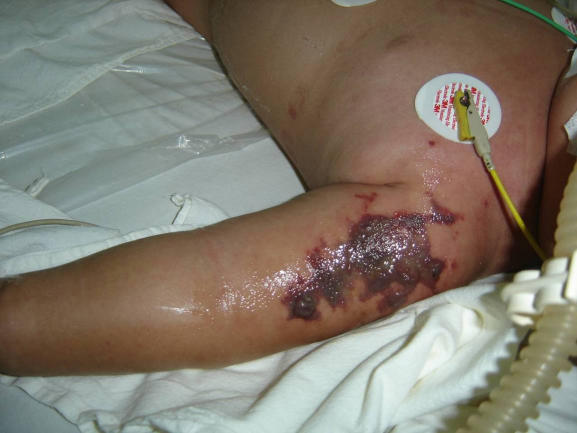
Rapidly progressive soft tissue infection after vaccination. Severe tissue necrosis around vaccination site in Child 4, two days following vaccination with MMR. MRSA was cultured from wound fluid.

The outbreak investigation led to the discovery of three additional children bringing the total to nine children, who required hospitalization for AEFI. All children were previously healthy and had been immunized in one of five different Health Centres in the same District between 28 April and 10 May 2006. Clinical and laboratory data on admission, and outcome of all children with AEFI are presented in Table 1. Three children presented with a rapidly progressive illness requiring respiratory and circulatory support, one of whom died and one of whom suffered severe neurological sequelae. Signs and symptoms in these children included high fever, regional or generalized rash or severe erythema, extensive and rapidly expanding swelling at the injection site, and shock, with raised creatine phosphokinase, serum creatinine, AST and ALT levels, all of which are compatible with toxic shock syndrome (TSS). Four children presented with skin and soft tissue infections, including one child (Child 4 in Table 1) with severe tissue necrosis requiring extensive surgical debridement which suggested the presence of necrotizing fasciitis (Figure 1). All skin and soft tissue infections developed at the vaccine injection site. Three children were admitted with an unspecific and relatively mild general illness (Table 1).

Six of the nine children received MMR vaccine in a single-dose lyophilized formulation with a single-dose diluent, two children received vaccinations of a two-dose liquid formulation of Hepatitis B vaccine, and the remaining child received a single dose lyophilized varicella vaccine. One of two nurses (vaccinator X or O) conducted the immunizations. All children with AEFI, except Child 9, were vaccinated by vaccinator X, who vaccinated a total of 185 children between 28 April and 10 May 2006, while vaccinator O vaccinated 39 children.

The investigation did not find any deficiencies or breaks in vaccine storage practices. Vaccines were prepared and, in the case of lyophilized vaccines, reconstituted immediately prior to administration, and delivered using single-use sterile syringe and needle. The injection site was swabbed with 70% isopropyl alcohol before injection. Infection control practices were found to be inadequate, including lack of hand washing facilities in at least one vaccination room visited. Vaccinator X indicated she routinely wore a single glove during vaccine reconstitution and administration, while vaccinator O used two gloves. Both vaccinators only changed their gloves after immunizing approximately three to six children and did not wash or disinfect their hands between children. Vaccinator X reported symptoms of a common cold in the days prior to 9 May 2006. She did not report any skin lesions and did not have skin lesions on inspection on 19 May 2006.

MRSA was isolated from pus from injection site abscesses in four children while blood cultures remained negative for all children. MRSA was also isolated from nose and throat swab samples from vaccinator X. Swab samples from mothers of Children 2, 4, and 9 were negative.

**Figure 2:**
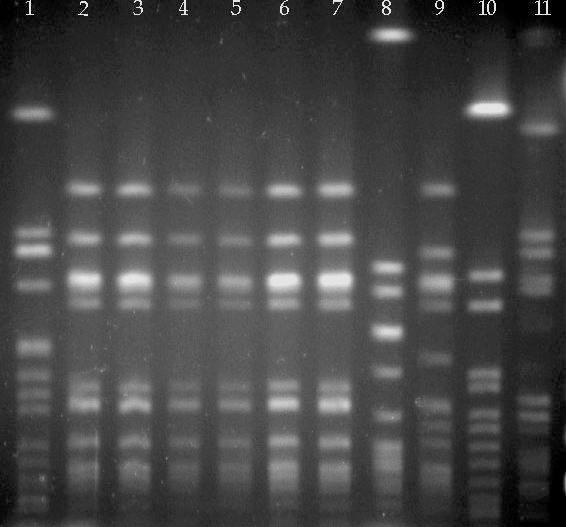
Molecular typing of MRSA isolates. Pulse-Field Gel Electrophoresis Patterns for MRSA strains isolated from four children vaccinated by vaccinator X (lanes 3–6 ), consecutive nasal swabs from vaccinator X (lanes 2, 7), a nasal swab from the son of vaccinator X (lane 8 ), strain HT2001-0751 (lane 9), hospital-acquired strain CV91 (lane 1) and strains ATCC 25923 (lane 10) and ATCC 33591 (lane 11).

All five MRSA isolates from four children and vaccinator X were resistant to oxacillin (MIC 2–4 mg/l), erythromycin and clindamycin but sensitive to all other drugs tested. The presence of the *mecA* gene was confirmed in all isolates. All five isolates had identical band patterns on PFGE analysis (Figure 2) and contained SCC*mec* cassettes type V (or type 5C). All strains were ST59 by MLST. PCR detected the presence of PV-*lukSF* and *seb.* On repeat testing on 23 June 2006, vaccinator X still carried the same strain of MRSA in here nose and throat (Figure 2) and her son also carried MRSA in his nose. Sensitivity patterns of the strains of vaccinator X and son were identical, but they had few bands in common on PFGE. The son's strain was PV-*lukSF* negative, SSC*mec* type IVa and a one allele variant of ST45, indicating that the isolates from vaccinator X and her son were unrelated. Further repeat nose and throat cultures from vaccinator X were collected on 7 September 2006 and were MRSA negative. Due to the unavailability of appropriate treatment, she remained untreated for her MRSA carriage until 7 September 2006.

ST59 is a relatively rare lineage of *S. aureus*, but CA-MRSA of this lineage has been reported in the USA [8], [22]. We compared a USA ST59 isolate HT2001-0751 with strain HCM3A, isolated from the nose of vaccinator X. On PFGE HCM3A showed five bands difference with HT2001-0751 (Figure 2). Microarray analysis of strains HCM3A and HT2001-0751 showed clustering of the core variable genes of both strains showing they belong to the same lineage and derive from a common ancestor (Table 2). However, there was substantial difference in MGE carriage, such as pathogenicity islands, SCC*mec*, and phage (Table 2). Thus the two strains are epidemiologically unrelated. Fully annotated microarray data have been deposited in BµG@Sbase (accession number: E-BUGS-45; http://bugs.sgul.ac.uk/E-BUGS-45) and ArrayExpress (accession number: E-BUGS-45).

**Table 2:** Key virulence and resistance genes in isolates HCM3A, HT2001 751, and MW2 as detected by microarray.

gene characteristic	*S. aureus* strain		
	HCM 3A	Ht 2001 751	MW2
core	*hla*	*hla*	*hla*
	*hlagACB*	*hlgACB*	*hlgACB*
	*hld*	*hld*	*hld*
	*clfAB*	*clfAB*	*clfAB*
lineage	ST59*	ST59	ST1
	*cap8*	*cap8*	*cap8*
	*sasG*	*sasG*	*sasG*
	*coa M0206v*	*coa M0206v*	*coa M0206v*
	*sasA* insert	*sasA* insert	*sasA* insert
	*sdrDE*	*sdrDE*	*sdrDE*
	-	-	*cna*
	*fnbpA R2580v*	*fnbpA R2580v*	*fnbpA N2291v*
	-	-	*lytN*
	*agr* class I	*agr* class I	*agr* class III
	*trap* R1926	trap R1926	*trap* M1775
	*sarT*	*sarT*	*sarT*
MGE**			
SCC*mec*	type V*	type IV*	type IV
	*mecA* *	*mecA, R1, ccrA2, B2*	*mecA, R1, ccrA2, B2*
	*far1*	-	-
phage	*PV-luk* * *^#^*	*PV-luk*	*PV-luk*
	*seg*	*seg*	*seg*
	-	-	*sak*
	-	-	*sea*
	-	-	*sek*
SaPI	*seb* *	*seb* *	-
	-	-	*sec*
plasmid	*blaZ*	*blaZ, I, R1*	*blaZ, I, R1*
	*cadDX*	*cadDX*	*cadDX*
	*arsB1,C*	*arsR1, B1, C,*	*arsR1, B1, C,*
	-	*bacteriocin* SAR0694-5	*bacteriocin* SAR0695

* Confirmed with PCR and/or sequencing

** Microarray data cannot conclusively prove the location of a gene. Assignation to a MGE is based on known location of that gene in other sequenced strains.

*hla*, α-haemolysin; *hlgACB*, γ-haemolysin; *hld*, δ-haemolysin; *clf*, clumping factors; ST, sequence type using MLST; *cap8*, capsule type 8; *sasG*, accumulation associated protein SA2285; *coa* M0206v, coagulase with four types described; *sasA*, haemagglutinin like gene which can contain an insert; *cna*, collagen binding protein; *sdr*, serine aspartate repeat proteins; *fnbpA*, fibronectin binding protein with three types described; *lytN*, cell wall hydrolase; *agr*, accessory gene regulator with 4 classes described; *trap*, target of RNAIII activating protein with 2 types described; *sarT*, staphylococcal accessory regulator T; MGE, mobile genetic element; SCC, staphylococcal cassette chromosome; *mec*, methicillin resistance; *ccr*, cassette chromosome recombinase with 3 types described; *far1*, putative fusidic acid resistance; *PV-luk*, Panton Valentine leukocidin; *seg*, enterotoxin G; *sak*, staphylokinase; *sea*, enterotoxin A; *sek*, enterotoxin K; *seb*, enterotoxin B; *sec*, enterotoxin C; *bla*, β-lactamase; *cad*, cadmium resistance; *ars*, arsenic resistance; *bacteriocin*, putative bacteriocin gene SAR0694-5; - denotes gene not detected; #, phage unrelated to PV-*luk* phage in MW2.

## Discussion

We describe an outbreak of severe infections caused by CA-MRSA acquired during routine vaccination injection. To our knowledge this is the first CA-MRSA outbreak associated with vaccination and linked to transmission by an asymptomatic health care worker. The current outbreak is also different to previously described clusters of CA-MRSA infections, as effective transmission of CA-MRSA occurred during an extremely short exposure period, i.e. during a single injection. Since the time of injection was documented, this outbreak demonstrates the short incubation period of severe disease caused by CA-MRSA. It also demonstrates that a single strain of CA-MRSA is capable of causing a spectrum of disease in otherwise healthy children, ranging from fatal toxic shock syndrome, necrotizing soft tissue infection, to abscesses at the injection site.

While bacterial infection associated with vaccination is occasionally reported, severe infections and deaths are uncommon. One case of toxic shock syndrome caused by *Streptococcus pyogenes* associated with vaccination by an asymptomatic colonized surgeon has been reported [23]. A review of AEFI in the US found only 250 (0.2%) reports of bacterial infection and 318 (0.2%) injection site abscesses among 128,717 adverse events reported during 1999–2001 when an estimated >1.9 billion vaccinations were administered [24]. The incidence of such events is likely to be variable in different settings and related to infection control practices as well as reporting practices. Severe bacterial infections, including TSS and soft tissue infections, have been linked with administration of multi-dose lyophilized vaccines [25]. However, in this outbreak the infections resulted from administration of single-dose lyophilized or two-dose liquid vaccines.

The results of the investigations suggested all children were infected by a common source. At the start of the outbreak a single MMR vaccine product was considered a potential source of infection. However the subsequent testing of that vaccine and the outcome of the investigation ruled out contamination. We did not study MRSA carriage in the affected children. However, the negative swab samples from the children's mothers suggest the circulation of strain HCM3A in different households, followed by autologous infection during vaccination, was unlikely. Cases of sepsis syndrome and rapidly progressive and fatal pneumonia caused by PVL-positive CA-MRSA, have been described in children worldwide [26] [4], [27], [28]. Child 6, 7 and 9 presented with relatively mild illness without localized symptoms. Unfortunately, additional diagnostic specimens were not obtained which precluded exclusion of other etiologies of AEFI.

Vaccinator X, with 17 years of prior experience as a vaccinator, was found to be a healthy transient carrier of CA-MRSA strain HCM3A who transmitted the strain during a two week period in which she performed vaccinations. None of her household members carried the same strain yet the transmission to the affected children occurred very efficiently during an extremely short period of contact. The vaccinator reported symptoms of a common cold during this period and the presence of an upper respiratory viral infection may have resulted in increased shedding of the carriage strain from nose and throat [29].

Insufficient hand hygiene during vaccine handling may have further contributed to the transmission of CA- MRSA. Current WHO recommendations do not require swabbing the injection site with isopropyl alcohol prior to vaccination unless the skin is visibly soiled or dirty [30]. However, the vaccinator reported that she routinely did this. Healthy carriers can transmit *S. aureus* by shedding the bacteria from the nose or other carriage sites into the environment and the air [31]. While determination of the precise route of transmission is impossible, we hypothesize that shedding of CA-MRSA, possibly increased due to the presence of a concurrent upper respiratory tract infection, may have resulted in contamination of the vaccine or the needle during vaccine preparation, or of the skin surface after disinfection. Our investigation highlights the potential risks of transmission of CA-MRSA and the importance of appropriate infection control measures, even during a short routine procedure such as injection in an out-patient setting. This investigation, for which all the work, with the exception of the microarray data, was undertaken in Ho Chi Minh City, highlights the benefits of having rapidly available multidisciplinary expertise at the site, capable of conducting detailed clinical, epidemiological, and molecular analysis of the outbreak and the causative microorganism.

Strain HCM3A showed all the characteristics of a CA-MRSA strain with low level oxacillin resistance that can easily be missed on testing, susceptibility to most other agents tested, and the presence of SCC*mec* type V. It contained several virulence factors that are associated with severe infections caused by *S. aureus*, including PV-*luk* and *seb*, on MGE [32], [33]. HCM3A belonged to the lineage ST59, which has also been described as causing CA-MRSA outbreaks in the USA, and strains of this lineage have been identified in the Netherlands, Singapore and Taiwan [10], [34](www.mlst.net) suggesting this lineage is spreading around the world. However, HCM3A carries many variant MGE compared to the USA strain and therefore is likely to be epidemiologically unrelated. It is likely that HCM3A evolved independently from methicillin-sensitive ST59 *S. aureus*, which acquired SCC*mec* and a range of virulence MGE. This confirms earlier observations that strains belonging to the same lineage as determined by MLST, can evolve significantly from each other [7], [35]. The spectrum of disease caused by the single strain HCM3A was varied and likely to be influenced by host factors. Similarly, other well characterised CA-MRSA strains, including the two from which the whole genome sequence has been determined, have caused a spectrum of disease. Strain MW2 (USA400, ST1) [36], which is included in our microarray, was the prototype CA-MRSA while strain USA300 (ST8) [37] has reached epidemic proportions in the United States [6]. Each belong to different lineages, yet can cause a similar range of diseases, emphasizing the key role of host factors in determining clinical presentation.

This outbreak is the first report of severe infections due to CA-MRSA in Viet Nam. It had a negative impact on vaccine uptake and resulted in a temporary nationwide suspension of MMR vaccination. The results of the investigation suggest that insufficient attention to hand hygiene may have led to transmission of CA-MRSA. Given the increasing prevalence of CA-MRSA worldwide, adherence to effective infection control strategies is paramount to prevent transmission of CA-MRSA and further outbreaks.
